# Multi objective optimization of AWJM parameters for ZrO_2_ coated MWCNTs reinforced HDPE nanocomposites using Taguchi Grey relational analysis

**DOI:** 10.1038/s41598-025-28887-6

**Published:** 2025-12-29

**Authors:** K. Suhas, B. R. N. Murthy, Anupama Hiremath, Pavan Hiremath

**Affiliations:** Department of Mechanical and Industrial Engineering, Manipal Institute of Technology, Manipal Academy of Higher Education (MAHE), Manipal, 576104 Karnataka India

**Keywords:** Polymer nanocomposites, Grey relational analysis (GRA), Surface roughness (R_a_), Kerf taper, Material removal rate (MRR), Multi-objective optimization, Engineering, Materials science

## Abstract

The machinability of High-Density Polyethylene (HDPE) reinforced with zirconia (ZrO_2_)-coated MWCNTs via Abresive Waterjet Machining (AWJM) was investigated in this study. The composite was fabricated with 3 wt% ZrO_2_-MWCNTs to enhance mechanical and thermal properties. A Taguchi L_9_ orthogonal array was employed to evaluate the effects of waterjet pressure, traverse speed, and stand-off distance on surface roughness (Ra), kerf taper (KT), and material removal rate (MRR). Grey Relational Analysis (GRA) was applied for multi-objective optimization, integrating the individual responses into a single Grey Relational Grade (GRG). Results indicated that lower traverse speed (100 mm/min) minimized R_a_ (4.286 μm), while higher pressure (200 MPa) and intermediate speed (150 mm/min) reduced kerf taper (0.04727 radians). The optimal parameter combination yielded the highest GRG (0.7361), balancing superior surface finish, dimensional accuracy, and machining efficiency. This study provides critical insights into precision AWJM of polymer nanocomposites for high-performance engineering applications.

## Introduction

The rapid advancement of polymer-based composite materials has revolutionized various industrial sectors due to their exceptional mechanical, thermal, and chemical properties. High-Density Polyethylene (HDPE), a widely used thermoplastic polymer, exhibits excellent chemical resistance, low moisture absorption, and good impact strength^1,2^. However, for applications demanding superior mechanical performance, HDPE is often reinforced with nanofillers to enhance its functional properties^3,4^. Among the variety of reinforcements, Multi-Walled Carbon Nanotubes (MWCNTs) have earned a significant attention due to their excellent mechanical, electrical, and thermal characteristics^5,6^. In particular, to enhance the full potential of MWCNTs in polymer composites, surface modification techniques are employed to improve their dispersion and interfacial adhesion with the polymer matrix^7,8^. Techniques such as acid treatment introduce carboxyl and hydroxyl groups to increase hydrophilicity, while silane coupling agents form covalent bonds for improved compatibility^8,9^. Other methods include plasma treatment, polymer grafting, and the deposition of metallic or ceramic nanoparticles like alumina (Al_2_O_3_), titania (TiO_2_), and zirconia (ZrO_2_)^10–12^. Among these, ZrO_2_ coatings are particularly effective in enhancing mechanical strength, thermal stability, and dispersion of MWCNTs in the HDPE matrix, while also reducing agglomeration and making them suitable for high-performance structural and thermal applications^13,14^.

ZrO_2_-coated MWCNTs offer improved compatibility with polymer matrices, owing to their surface functionality, which promotes better stress transfer and uniform distribution within the HDPE matrix^14^. This synergy significantly enhances the composite’s mechanical robustness, making it a potential candidate for high-performance engineering applications. The integration of ZrO_2_-coated MWCNTs into HDPE matrices not only augments the composite’s structural properties but also influences its machinability, especially under unconventional machining processes such as Abrasive Water Jet Machining (AWJM)^15^.

AWJM is a non-traditional, versatile machining process widely employed for cutting difficult-to-machine materials. It utilizes a high-pressure stream of water mixed with abrasive particles to erode material from the workpiece^16,17^. AWJM presents several advantages over conventional machining methods, such as minimal thermal distortion, negligible tool wear, and the ability to machine complex shapes. However, the quality of machining outcomes is influenced by several process parameters, including waterjet pressure, traverse speed, and stand-off distance (SOD). Accurate control and optimization of these parameters are crucial for achieving desired surface integrity and dimensional accuracy^18,19^.

In this context when machining HDPE reinforced with ZrO₂-coated MWCNTs, it is important to clearly understand how the machining settings affect the final output. The primary performance indicators in AWJM include surface roughness (R_a_), kerf width (KW), and material removal rate (MRR)^20^. Surface roughness reflects the surface integrity of the machined part, kerf width determines the dimensional precision of the cut, and MRR indicates the efficiency of the machining process^21^. These output responses are intricately affected by the input machining parameters and the composite material’s intrinsic properties^22^. Given the complexity of the AWJM process and the interdependent nature of the influencing parameters, robust experimental design and multi-objective optimization techniques become essential^23^. The Taguchi method, a statistical approach based on orthogonal arrays, is widely used for process optimization due to its simplicity, cost-effectiveness, and ability to identify significant factors influencing the process^24^. By employing the Taguchi technique, optimal parameter settings can be determined with a minimal number of experiments, ensuring efficient use of resources^16^.

The study conducted by Doğankaya et al.^25^ investigated the AWJM of ultra-high molecular weight polyethylene (UHMWPE) to assess the influence of water pressure, traverse speed, stand-off distance, and abrasive flow rate on surface roughness, kerf angle, and dimensional accuracy using RSM, ANOVA, and PSO. Higher water pressure and abrasive flow improved surface finish, while increased traverse speed and nozzle distance worsened it. Dimensional errors and delamination were mainly attributed to abrasive entrapment between thin UHMWPE layers. Another study on AWJM drilling of UHMWPE employed a full-factorial design varying water jet pressure (210–360 MPa), abrasive flow (250–400 g/min), and traverse speed (150–600 mm/min), revealing that higher pressure and lower speed yielded smoother surfaces and improved penetration.

Müller et al.^26^ compared AWJM and non-abrasive WJ cutting of polypropylene (PP) and PVC-U coated with polyurethane and acrylic layers. At constant pressure (380 MPa), the traverse speed (50–1000 mm/min) significantly influenced kerf taper and surface quality, with PP–MOBIHEL showing the lowest taper angle (0.09°). Similarly by Sampath Boopathi et al.^27^ studied AWJM of neem wood–PP composites using Taguchi optimization, finding that neem powder and water pressure had the strongest effect on kerf angle and surface roughness, with optimal parameters (20% NW, 2% talc, 450 mm/min, 1000 bar) achieving KA = 0.96° and SR = 8.12 μm. S Muralidharan et al.^28^ analyzed AWJM of E-glass/PP composites using L16 Taguchi and GRA methods, where abrasive flow rate most influenced circularity (43.25%) and optimal settings were 80 g/min, 4 mm SOD, and 400 mm/min.

Ganesan et al.^17^ examined AWJM of 3D-printed Onyx (nylon–carbon composite) using Taguchi, ANOVA, GA, and MFO approaches to minimize delamination and roughness, identifying 450 g/min abrasive flow, 30 mm/min traverse speed, and 12 mm drilling diameter as optimal. Vasanthkumar et al. in^29^ studied AWJM of Nylon-6 reinforced with 15 wt% seashell biofiller via Taguchi’s L18 design, reporting that higher pressure, smaller stand-off distance, and finer garnet particles (75 μm) improved MRR and surface finish while minimizing kerf angle.

However, in multi-response optimization scenarios, where multiple output parameters need to be simultaneously optimized, conventional Taguchi analysis might not yield comprehensive insights. To address this limitation, Grey Relational Analysis (GRA) is employed^30^. GRA is a powerful decision-making tool that converts multiple performance characteristics into a single grey relational grade, facilitating the simultaneous optimization of all responses^31,32^. The integration of Taguchi design with GRA provides a robust framework for optimizing AWJM parameters of HDPE reinforced with ZrO_2_-coated MWCNTs.

In this study, an attempt is made to investigate the AWJM characteristics of HDPE reinforced with ZrO_2_-coated MWCNTs. The composite material is fabricated with an optimal dispersion of 3 wt% ZrO_2_-coated MWCNTs to achieve improved mechanical and thermal stability. The AWJM process is analyzed under varying levels of waterjet pressure, stand-off distance, and traverse speed. A Taguchi L9 orthogonal array is employed to design the experiments, considering three levels of each input parameter. The machining performance is evaluated in terms of surface roughness (Ra), kerf taper (KT), and material removal rate (MRR). The experimental data are subjected to statistical analysis using signal-to-noise (S/N) ratios to identify the significant machining parameters influencing each output response. Subsequently, GRA is applied to perform a multi-response optimization by converting the S/N ratios of individual responses into a single Grey Relational Grade (GRG). This approach allows the identification of the optimal combination of machining parameters that ensures minimal surface roughness and kerf width while maximizing the material removal rate. The findings from this study are expected to provide valuable insights into the machinability of HDPE/ZrO_2_-MWCNT nanocomposites and contribute to the development of efficient, high-precision machining strategies for advanced polymer-based materials.

## Materials and fabrication

### Materials

High-density polyethylene (HDPE) 002DP48, procured in pellet form from Indian Oil Propel, Mumbai, India, is a PE100 grade material. MWCNTs with 99% purity were sourced from AD-Nano Technologies Pvt. Ltd. Essential chemicals such as 98% ethanol, 70% nitric acid (HNO_3_), and polytetrafluoroethylene (PTFE) filter membranes with a pore size of 0.5 μm were obtained from Raman lab equipment’s Pvt. Ltd., Karnataka, India. Additionally, zirconium oxychloride octahydrate (ZrOCl_2_·8H_2_O) was acquired from Loba Chemie, India, other essential materials are sourced locally.

### Preparation of zirconia coated MWCNT

#### Acid functionalization of MWCNT

To minimize the agglomeration of MWCNTs and to achieve superior coating of zirconia (ZrO_2_), MWCNTs are subjected to acid treatment using various acid combinations. According to a study by Suhas K et al.,^13^ MWCNTs treated with 8 M HNO_3_ at 60 °C for 3 h exhibit stability with minimal agglomeration and improved ZrO_2_ coating. Based on these findings, the MWCNTs undergo acid treatment using a reflux setup, which prevents acid loss during evaporation.

To prepare an 8 M HNO_3_ solution, 100 ml of deionized water was used. Subsequently, 1 g of MWCNTs was precisely weighed and introduced into the solution. The mixture was subjected to magnetic stirring for 1 h to facilitate MWCNTs dispersion within the acid solution.

Before the acid treatment, the solution was subjected to ultrasonication for an hour to de-agglomerate the MWCNTs, facilitating uniform dispersion. To prevent temperature, increase during ultrasonication, the ultrasonic bath was maintained at ambient temperature, with ice cubes introduced to moderate temperature rise^33^. The mixture was then refluxed for 3 h at 60℃ with simultaneous magnetic stirring. Temperature and reflux duration are critical parameters higher temperatures and extended durations can degrade MWCNTs, causing weight loss, wall ruptures, and shortening of nanotubes^34^.

After completing the reaction, the mixture was allowed to cool to room temperature. The mixture was filtered using a PTFE filter membrane and thoroughly washed multiple times with deionized water to remove residual acid. The pH was monitored and adjusted to a range of 6–7 through repeated washing. The MWCNTs were dried overnight in a hot air oven at 60 °C to ensure evaporation of moisture content. The resulting functionalized MWCNTs (f-MWCNTs), initially in cake form, were crushed into a powder using a mortar and pestle.

#### Coating of ZrO_2_ on MWCNT using hydrothermal setup

**Fig. 1:**
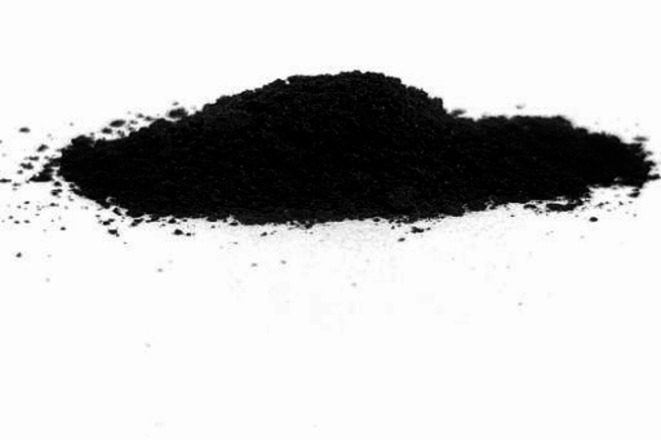
ZrO_2_ coated MWCNTs.

Zirconium oxychloride octahydrate (ZrOCl_2_·8H_2_O) is used as a precursor to create ZrO_2_ coatings on f-MWCNTs. The synthesis technique consists of a series of regulated procedures to produce a homogeneous and well-dispersed MWCNT coating. 0.3 M aqueous solution ZrOCl_2_·8H_2_O is prepared using 100 ml deionized water and subsequently, 100 mg of f-MWCNTs is accurately weighed and introduced into the ZrOCl_2_·8H_2_O solution. This mixture is vigorously stirred using a magnetic stirrer for 30 min to ensure thorough mixing. The solution is ultrasonicated in a water bath for 1 h to improve the dispersion of f-MWCNTs in the ZrOCl_2_·8H_2_O solution, resulting in a dark, viscous liquid with uniformly distributed MWCNTs. The resultant black suspension is then transferred to a stainless-steel Teflon-lined autoclave, where it is maintained at 200 °C for 18 h to facilitate the hydrothermal reaction. After the hydrothermal process, the autoclave is allowed to cool naturally to room temperature. The solution is then subjected to a drying process in a hot air oven at 60 °C until complete evaporation of moisture. The final product is a light black powder, identified as ZrO_2_-coated MWCNTs as seen in Fig. 1.

### Fabrication of ZrO_2_-coated MWCNTs reinforced HDPE composite

The HDPE pellets were pulverized into a powdered form using a ROTO pulveriser. The primary objective of grinding the pellets was to achieve better mixing with zirconia-coated MWCNTs. The HDPE/ZrO_2_ coated MWCNTs blends were obtained via a twin-screw extruder (O-Micron 10 Twin Screw Extruder, Steer Engineering, India). This ensures thorough dispersion of ZrO_2_ coated MWCNTs and uniform distribution of materials, resulting in consistent blending of HDPE with ZrO_2_ coated MWCNTs.

In the current work, we have considered 3 weight% of zirconia coated MWCNTs and is represented as ZMH3. In our previous work for 3 wt% has got the maximum tensile strength and hardness so we have considered this wt% for AWJM^14^. Later, powdered HDPE were preheated at 60 °C for 1 h to remove moisture and different weight percentages of ZrO_2_ coated MWCNTs were weighed and manually mixed for 10 min in a plastic container in order to obtain uniform distribution of HDPE and ZrO_2_ coated MWCNTs.

Then the mixture is fed into the barrel of the extruder from a hopper. The barrel of the twin-screw extruder is divided into three distinct heating zones. The feed zone is maintained at 180 °C, ensuring optimal preheating and softening of the raw material. The melting zone operates at 200 °C, facilitating efficient melting and homogenization of the polymer. Finally, the discharge zone is maintained at 160 °C, allowing proper cooling and stabilization of the molten polymer before exiting through the die. The screw rotates at 60 RPM, ensuring consistent material flow and effective processing^14^.

**Fig. 2:**
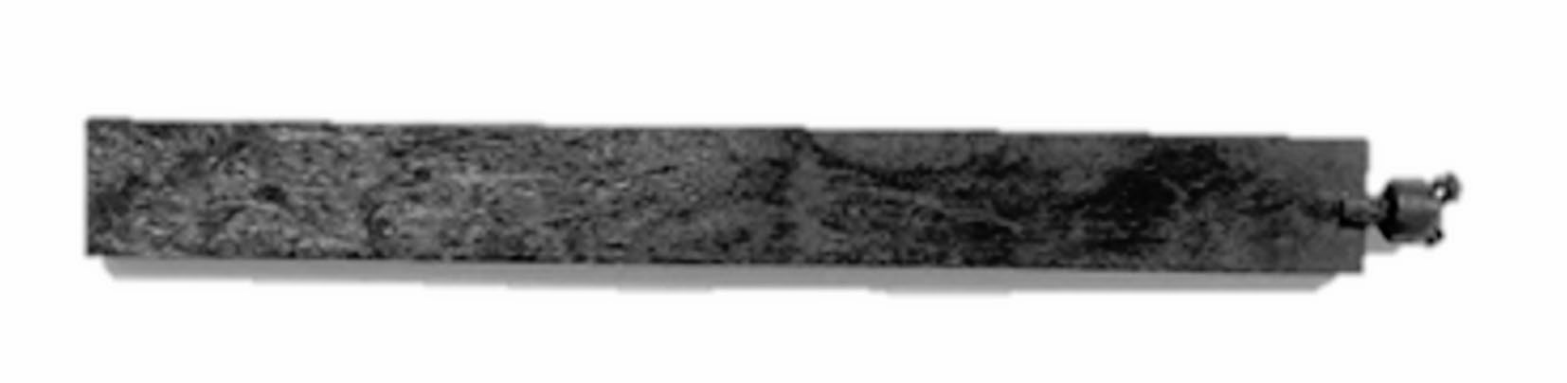
Fabricated test specimen for AWJM.

The composite specimens were fabricated using the injection moulding technique, barrel temperature was maintained at 250 °C, ensuring proper melting, flow, and moulding of the polymer composite materials. The fabricated composite specimens were subjected to mechanical characterization, including tensile, flexural, impact, and hardness testing, across varying weight fractions (1, 2, 3, and 4 wt%) of ZrO₂-coated MWCNTs, as detailed in Table 1. The experimental procedures and comprehensive results are elaborated in our earlier studies^14^. Based on the superior tensile and impact strength exhibited by the 3 wt% formulation, it was selected for further investigation in the current study involving AWJM. AWJM was conducted on composite sample with a nominal thickness of 3.2 mm, as depicted in Fig. 2.

**Table 1:** Mechanical properties of HDPE and ZrO₂-MWCNT/HDPE composites.

Sample	Tensile strength (MPa)	Flexural strength (MPa)	Impact strength (kJ/m^2^)	Hardness (VHN)
H0	25.99 ± 1.2	36.45 ± 0.7	7.2 ± 0.8	19.5 ± 2.1
ZMH1	30.64 ± 0.9	38.13 ± 0.9	8.4 ± 0.6	21.1 ± 1.7
ZMH2	33.57 ± 1.5	39.81 ± 1.1	9.1 ± 0.9	24.3 ± 1.4
ZMH3	38.90 ± 1.1	40.49 ± 1.1	11.6 ± 1.2	27.7 ± 1.8
ZMH4	35.99 ± 0.8	41.26 ± 1.3	10.8 ± 1	31.4 ± 1.2

### Abrasive water jet machining (AWJM)

**Fig. 3:**
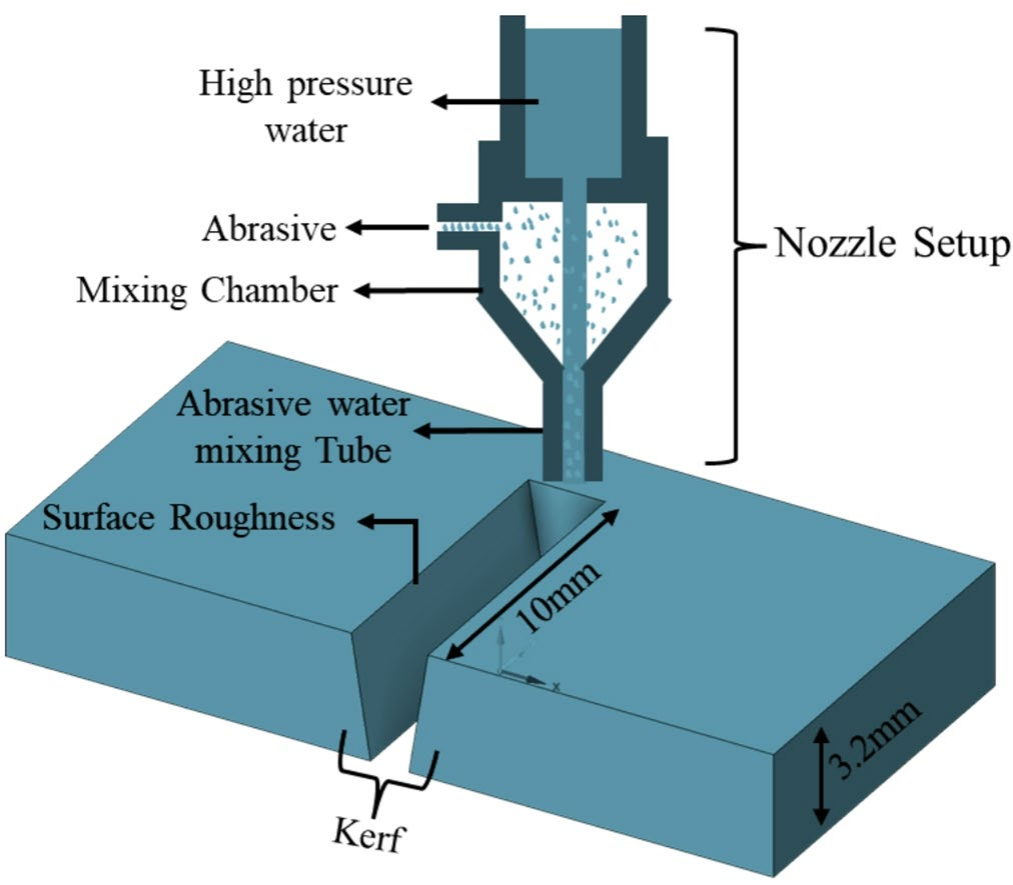
Abrasive waterjet machining schematic representation.

The composite samples were machined using a Maxiem 1515 (injection-type) AWJ machine from OMAX Corporation. The machine was equipped with a 30 HP direct drive pump for the cutting experiments, as shown in Fig. 3. The machining process was conducted on injection moulded samples with a thickness of 3.2 mm and a machining distance of 10 mm for all samples.

AWJM process involves several input parameters that need to be adjusted to achieve both good quality and efficiency. However, considering all these parameters can be time-consuming and may create confusion when evaluating cutting performance. The shape and quality of the cut depend greatly on selecting the right process settings. In this study, three important input parameters waterjet pressure (P), traverse speed (V), and standoff distance (SOD) were chosen, each tested at three different levels. These parameters were selected based on a review of previous studies and the machine’s specifications, with both dynamic and constant parameters some of the constants considered in this study are Garnet size, Mass flow rate (Mf), Orifice diameter and Angle of target. Selected parameters and their levels are shown in Table 2. There are several types of abrasives used in the AWJM process, including garnet, olive sand, steel grit, silicon carbide (SiC), silica sand, aluminium oxide (Al_2_O_3_), and glass beads^35^. Among these, garnet has shown the best performance due to its sharp edges and good flow properties, which make it more effective at cutting, and it is also affordable.

**Table 2:** Variable and constant machining parameters.

	Units	Leve1	Level2	Leve3
Dynamic parameters				
Waterjet pressure (p)	MPa	100	150	200
Traverse speed (V)	mm min^− 1^	100	150	200
Standoff distance (SOD)	mm	2	3	4
Constant parameters				
Garnet size	MESH	60		
Mass flow rate (Mf)	g min^− 1^	100		
Orifice diameter	mm	0.35		
Angle of target		90°		

### Design of experiments

To systematically investigate the influence of process parameters on the machining performance of HDPE/ZrO₂-MWCNT nanocomposites, the Taguchi method was employed using an L9 orthogonal array. This experimental design was chosen due to its efficiency in analyzing multiple factors with a reduced number of experimental trials as shown in Table 3. Three key input parameters waterjet pressure, stand-off distance, and traverse speed were each varied at three levels. The waterjet pressure, a critical factor influencing the kinetic energy of abrasive particles, was varied at three levels: 100 MPa, 150 MPa, and 200 MPa. Higher pressures are generally associated with enhanced material removal rates and narrower kerf widths due to increased jet penetration capabilities. Traverse speed was also varied at three levels: 100 mm/min, 150 mm/min, and 200 mm/min. This parameter significantly affects the exposure time of the jet on the material surface, thereby influencing both the depth of cut and surface finish. The stand-off distance, which is the gap between the nozzle tip and the target material, was adjusted at 2 mm, 3 mm, and 4 mm. The SOD plays a vital role in controlling the focus and dispersion of the abrasive jet; smaller distances often result in more concentrated impact energy and finer surface finishes.

In addition to the dynamic parameters, several constant parameters were maintained throughout all trials to ensure consistency and isolate the effects of the primary input variables. These included a garnet abrasive size of 60 mesh, a mass flow rate (Mf) of 100 g/min, an orifice diameter of 0.35 mm, and a fixed impingement angle of 90°, ensuring perpendicular impact of the abrasive jet on the work surface. These settings were selected based on preliminary trials and literature to provide effective material erosion while minimizing experimental variability.

The L9 array enabled the evaluation of the main effects of these parameters on three critical output responses: surface roughness (R_a_), kerf taper (KT), and material removal rate (MRR). Each experiment was conducted once under identical conditions, and the respective responses were measured and recorded for analysis. The results obtained from these trials were further used to perform signal-to-noise (S/N) ratio analysis to determine the significance and contribution of each parameter.

**Table 3:** Experimental design using L9 orthogonal array.

Experiment No	Process parameters		
	Waterjet pressure (*P*) MPa	Traverse speed (V) mm min^− 1^	Standoff distance (SOD) mm
1	100	100	2
2	100	150	3
3	100	200	4
4	150	100	3
5	150	150	4
6	150	200	2
7	200	100	4
8	200	150	2
9	200	200	3

### Surface roughness measurement

**Fig. 4:**
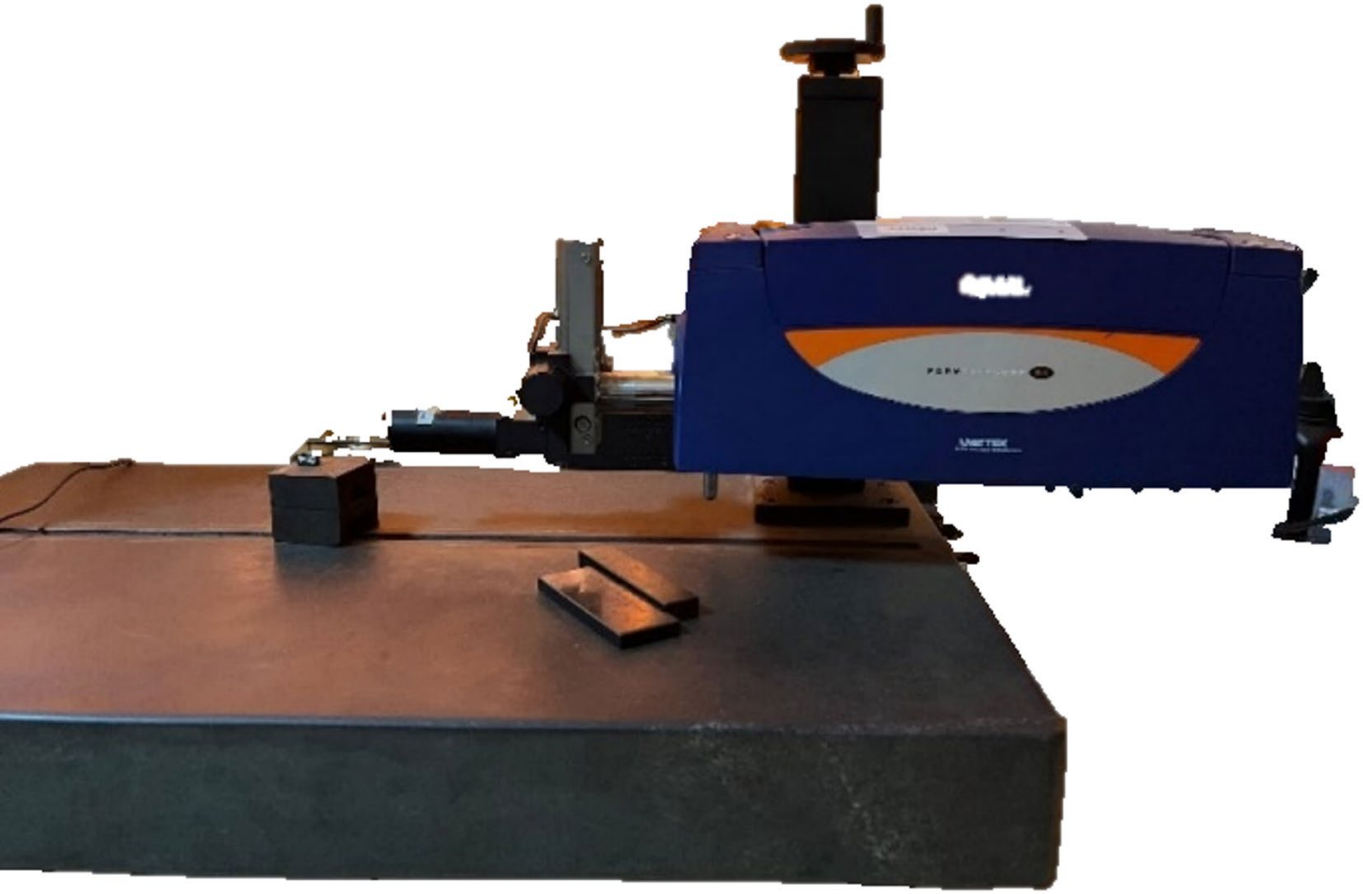
Taylor Hobson surface tester.

**Fig. 5:**
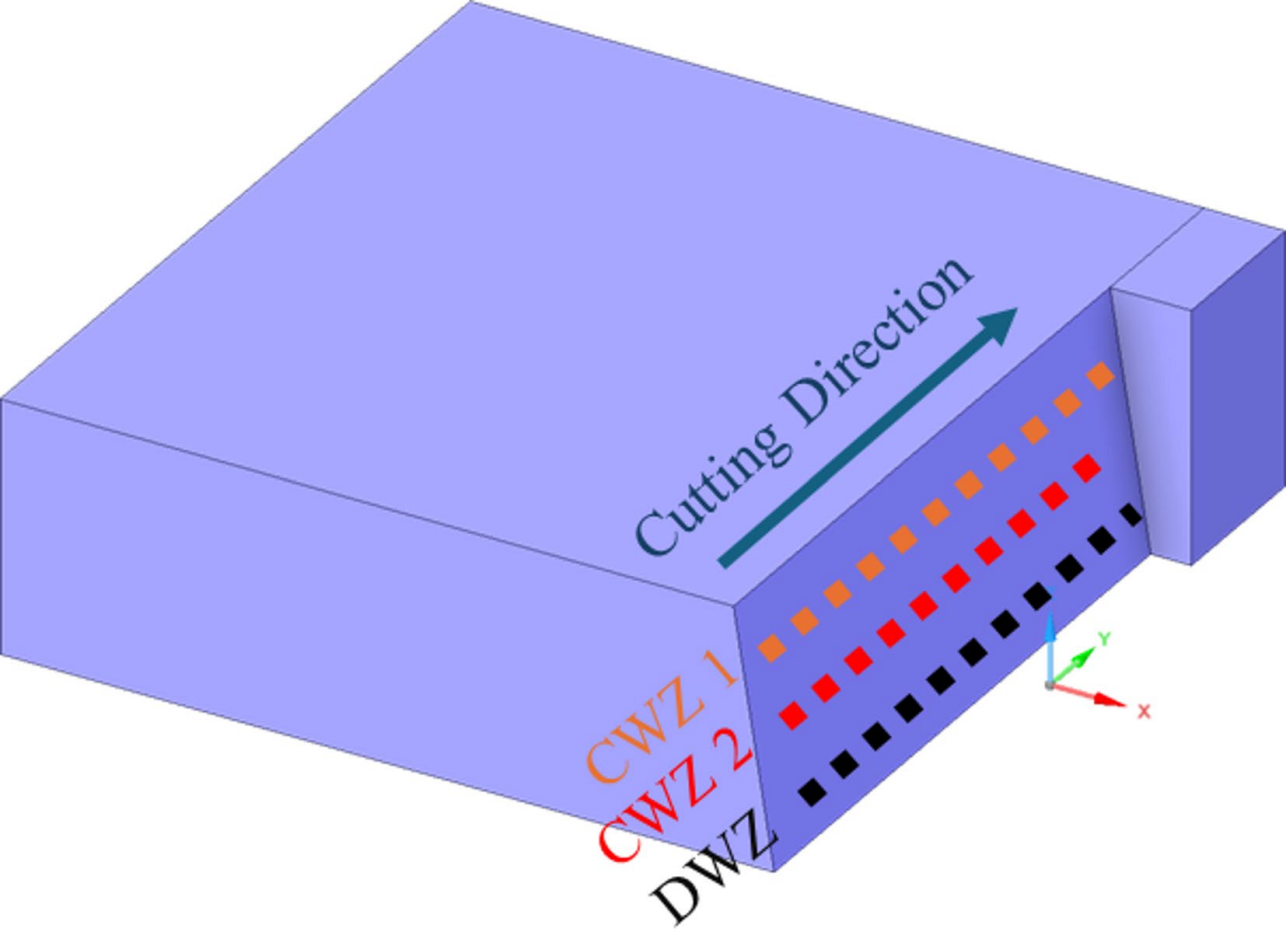
Surface roughness measuring regions.

Surface roughness was measured using a Taylor Hobson surface tester (Fig. 4) with a tip radius of 2.5 μm and an evaluation length of 10 mm was used as per ISO 4287. Roughness is evaluated on both the Cutting Wear Zone (CWZ) and the Deformation Wear Zone (DWZ), as depicted in Fig. 5. Precisely, roughness measurements were conducted in two regions of the CWZ and one region of the DWZ. The CWZ is a striation-free zone, characterized by its smooth surface and superior surface finish^35^. In contrast, the DWZ exhibits striations, which are formed as a result of the waterjet impingement on the material’s surface. Each abrasive particle in the water jet acts as a cutting edge upon impact with the top surface. As the high-velocity water jet removes material from the top surface, the water jet velocity decreases when it penetrates the material. Consequently, the interaction between the eroded material and the abrasive particles leads to the formation of striation marks at the lower end of the specimen. The average value of three R_a_ measured were considered for further analysis.

### Kerf width measurement

**Fig. 6:**
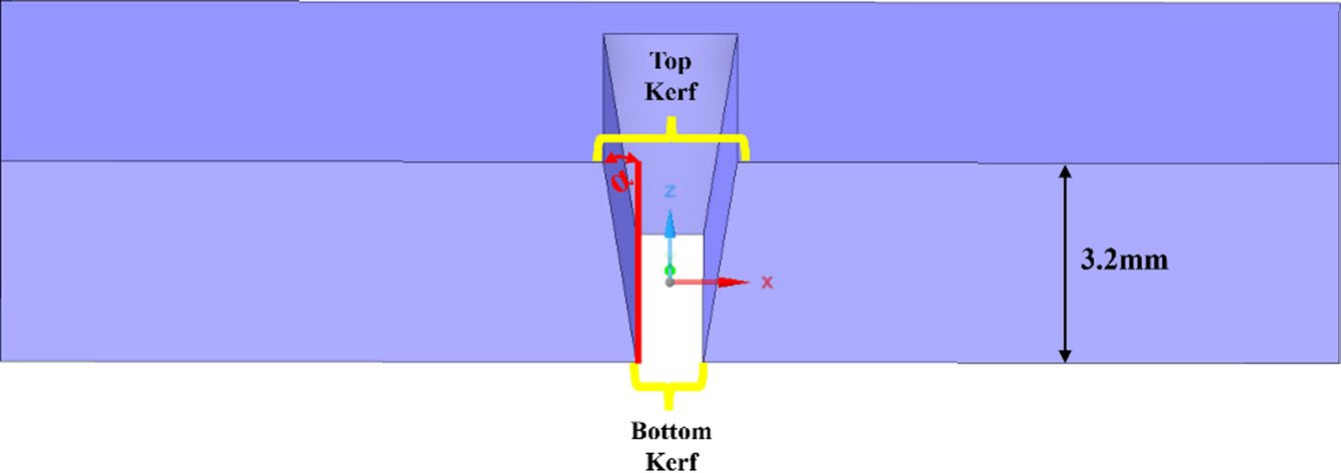
Schematic representation of kerf geometry.

Kerf width, a critical geometrical characteristic representing the width of material removed during AWJM was measured systematically using an optical microscope and dimensional analysis of the machined samples. Figure 6 illustrates the schematic representation of kerf geometry generated during AWJM of ZrO_2_ coated MWCNTs reinforced HDPE composites. As depicted in the image, the kerf produced by the abrasive waterjet exhibits a tapered profile, with a wider top kerf at the surface of the workpiece and a narrower bottom kerf at the exit. The total thickness of the machined specimen was maintained at 3.2 mm, ensuring uniformity across all samples.

**Fig. 7:**
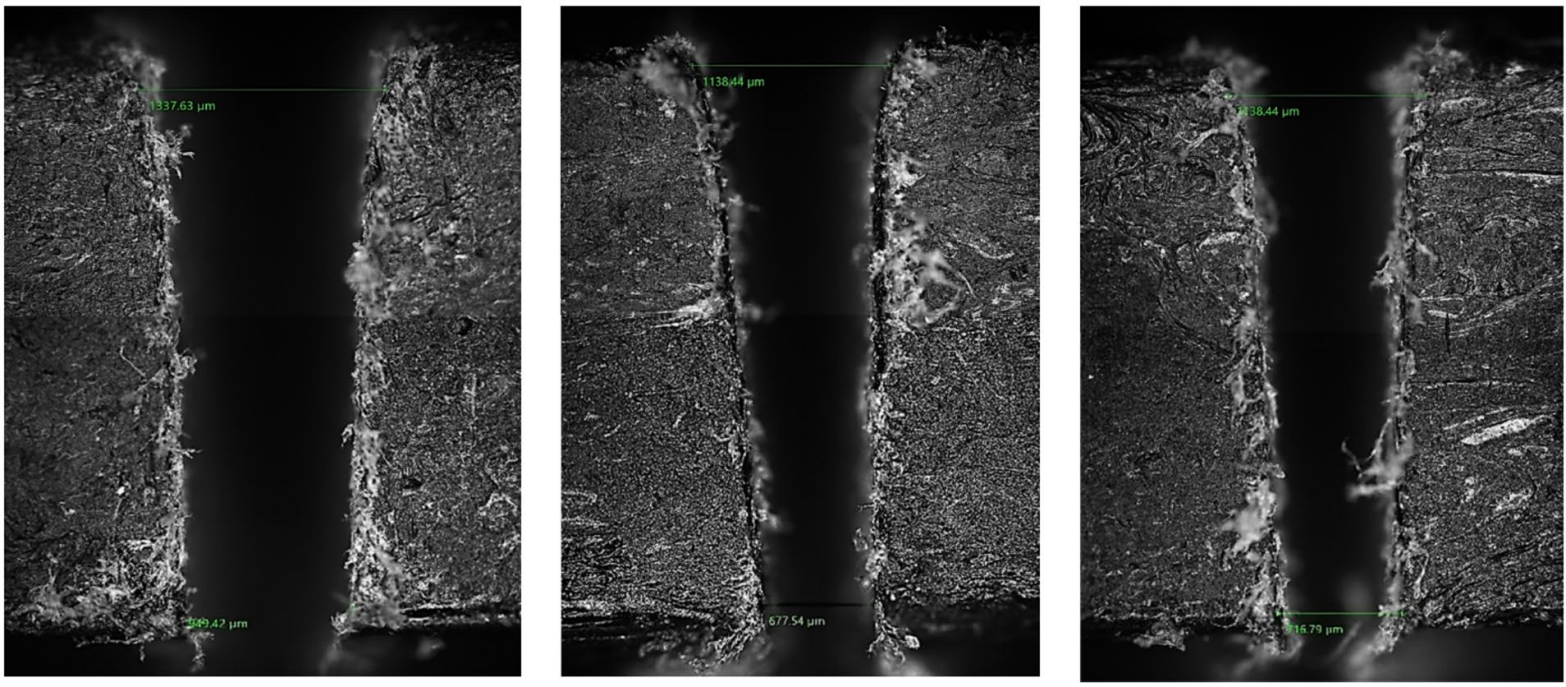
Kerf measurement using optical microscope.

The top kerf width refers to the maximum width of the groove created by the machining process at the entry surface of the work piece, where the abrasive jet first contacts the material. This width is measured using a calibrated optical microscope or a high-resolution image analysis tool to ensure an accurate depiction of the kerf boundary, as illustrated in Fig. 7. The bottom kerf width, on the other hand, is the narrower measurement found at the exit side of the jet. Precise measurements of this dimension are taken after sectioning the machined sample to expose the kerf profile and accurately capture its geometry.

**Average Kerf width (KA)**: The mean of the top and bottom kerf widths was calculated using Eq. (1)^21^ and used for further statistical analysis, as it represents the overall material removal in the lateral direction.

1 $$KA=\frac{{Top~kerf~width+Bottom~Kerf~Width~}}{2}$$

**Kerf taper angle (KT)**: The angular deviation of the sidewalls of the kerf from vertical (Z-axis) was calculated using Eq. (2)^21^ for further evaluation of dimensional accuracy and process stability.

2 $$KT={\tan ^{ - 1}}\left( {\frac{{Top~Kerf~width - Bottom~Kerf~width}}{{2t}}} \right)$$

### Material removal rate

Material Removal Rate (MRR) is a crucial metric for evaluating the efficiency of the Abrasive Water Jet Machining (AWJM) process. In this study, MRR was calculated based on the geometric volume of material removed during machining and the traverse speed of the cutting nozzle, as outlined in Eq. (3)^29^. The traverse speed was regulated using the CNC settings of the machine, and kerf widths were measured with a calibrated optical microscope. The resulting MRR values allowed for quantitative comparisons across various combinations of machining parameters.

3 $$MRR=KA \times d \times v$$

Where, $KA$ is the Average kerf width, *d* is the thickness of the work piece and *v* is the traverse speed.

### Response optimization using Taguchi

The Taguchi method was employed to optimize the AWJM parameters concerning multiple performance characteristics surface roughness (R_a_), kerf width, and material removal rate (MRR). An L9 orthogonal array was used to systematically design the experiments based on three input parameters: waterjet pressure, traverse speed, and stand-off distance, each at three levels.

To assess the performance of each parameter setting, the Signal-to-Noise (S/N) ratio was calculated using Taguchi’s quality criteria, which aim to improve robustness by minimizing variability in the response variables. The selection of the S/N ratio formulation depended on the nature of each performance characteristic.

- Smaller-the-Better: This criterion was applied for surface roughness (R_a_) and kerf width, where lower values indicate improved surface quality and dimensional precision. The corresponding S/N ratio is calculated as^36^.
  4 $$S/N=\left( { - 10 \cdot log10\left( {\frac{1}{n}\mathop \sum \limits_{{i=1}}^{n} y_{i}^{2}} \right)} \right)$$

- Larger-the-Better: This criterion was adopted for MRR, where higher values signify greater machining efficiency. The S/N ratio for this case is given by^37^.
  5 $$S/N=\left( { - 10 \cdot log10\left( {\frac{1}{n}\mathop \sum \limits_{{i=1}}^{n} \frac{1}{{y_{i}^{2}}}} \right)} \right)$$

### Multi response optimization using grey relation analysis (GRA)

To conduct multi-objective optimization in the AWJM of HDPE composites reinforced with ZrO₂-coated MWCNTs, Grey Relational Analysis was utilized to optimize multiple performance characteristics: surface roughness (R_a_), kerf width, and material removal rate (MRR). This method effectively combines these three critical output responses into a single Grey Relational Grade, which aids in identifying the optimal machining conditions. The steps involved in determining the Grey Relational Grade are as follows:

#### Data normalization

To make the output responses dimensionless and comparable, the experimental data for each response variable were normalized. Since the performance characteristics possess different units and scales, normalization was conducted to bring all data into a comparable range between 0 and 1. For R_a_ and kerf width, where lower values are desirable i.e., Smaller-the-Better, the normalized value was computed using Eq. (6), Where, ${x_i}\left( k \right)$ is normalized value for $i{\mathrm{th}}$ experiment $k{\mathrm{th}}$ response, the ${y_i}\left( k \right)$ is actual measured value from AWJM tests on ZrO₂-MWCNTs/HDPE composites.

6 $${x_i}\left( k \right)=\frac{{{\mathrm{max~}}{y_i}\left( k \right) - {y_i}\left( k \right)}}{{{\mathrm{max~}}{y_i}\left( k \right) - min{y_i}\left( k \right)}}$$

For MRR, where higher values are preferred i.e., Larger-the-Better, the normalization was performed using Eq. (7)

7 $${x_i}\left( k \right)=\frac{{{y_i} - ~~min{y_i}\left( k \right)}}{{{\mathrm{max~}}{y_i}\left( k \right) - min{y_i}\left( k \right)}}$$

#### Grey relational coefficient (GRC)

After normalization, the grey relational coefficient was calculated for each response to express its relative closeness to the ideal normalized value. The grey relational coefficient can be determined using Eq. (8) where, ${\Delta _i}\left( k \right)$ is difference between the ideal normalized value usually 1 and the actual normalized value, ${\Delta _{min}}$ and ${\Delta _{max}}$ minimum and maximum of all ${\Delta _i}\left( k \right)$ values across all trials and $\xi$ is distinguishing coefficient in this study its assumed as 0.5

8 $${\xi _i}\left( k \right)=\frac{{{\Delta _{min}}+\zeta \cdot {\Delta _{max}}}}{{{\Delta _i}\left( k \right)+\zeta \cdot {\Delta _{max}}}}$$

#### Grey relational grade (GRG)

To obtain a single performance index for each experiment, the GRG was calculated using Eq. (9) as the average of the GRCs across all three responses R_a_, kerf width, and MRR. Where, ${\gamma _i}$ Grey relational grade and ${\xi _i}\left( k \right)$ is GRC.

9 $${\gamma _i}=\frac{1}{3}\mathop \sum \limits_{{k=1}}^{3} {\xi _i}\left( k \right)$$

Once the GRG was calculated for each experimental run, the results were ranked in descending order to identify the optimal machining condition. A higher GRG value indicates a closer approximation to the ideal performance, which corresponds to low surface roughness and kerf width, and high MRR.

Each experiment was assigned a rank based on the GRG, with rank 1 corresponding to the highest GRG, i.e., the most favourable overall response. This ranking helped identify the optimal combination of waterjet pressure, traverse speed, and stand-off distance for machining ZrO₂ coated MWCNT reinforced HDPE composites.

## Results and discussions

### Effect of process parameters on surface roughness (R_a_)

The Table 4 represents the responses for all the L_9_ orthogonal array experiments. Surface roughness is an essential indicator of surface integrity after machining, especially in AWJM. This measure is influenced by complex interactions involving jet coherence, particle momentum, and erosion mechanics. In this study, we analyzed surface finish using the signal-to-noise (S/N) ratio approach from Taguchi’s methodology as shown in Table 5. A less negative S/N ratio indicates improved surface quality, which translates to lower R_a_ values.

**Table 4:** Experimental design using L_9_ orthogonal array with responses.

Experiment no	Process parameters			Response parameters		
	Waterjet pressure (*P*) MPa	Traverse speed (V) mm min^− 1^	Standoff distance (SOD) mm	*R*_a_ (µm)	KT (radians)	MRR (mm^3^/min)
1	100	100	2	4.286	0.0773	48.25
2	100	150	3	4.388	0.0927	71.49
3	100	200	4	4.451	0.1228	109.10
4	150	100	3	4.468	0.1014	56.54
5	150	150	4	4.382	0.1221	86.96
6	150	200	2	4.411	0.0922	93.08
7	200	100	4	4.341	0.0922	55.24
8	200	150	2	4.312	0.04727	68.87
9	200	200	3	4.395	0.07472	91.13

**Table 5:** S/N ratio data.

Experiment no	S/*N* ratio		
	*R*_a_ (µm)	KT (radians)	MRR (mm^3^/min)
1	-12.641	22.24178	-33.6713
2	-12.8453	20.65521	-37.0849
3	-12.9692	18.21312	-40.7571
4	-13.0023	19.87832	-35.0472
5	-12.8334	18.26024	-38.787
6	-12.8907	20.69791	-39.3778
7	-12.7518	20.69782	-34.8457
8	-12.6936	26.50717	-36.7615
9	-12.8592	22.53054	-39.1939

**Fig. 8:**
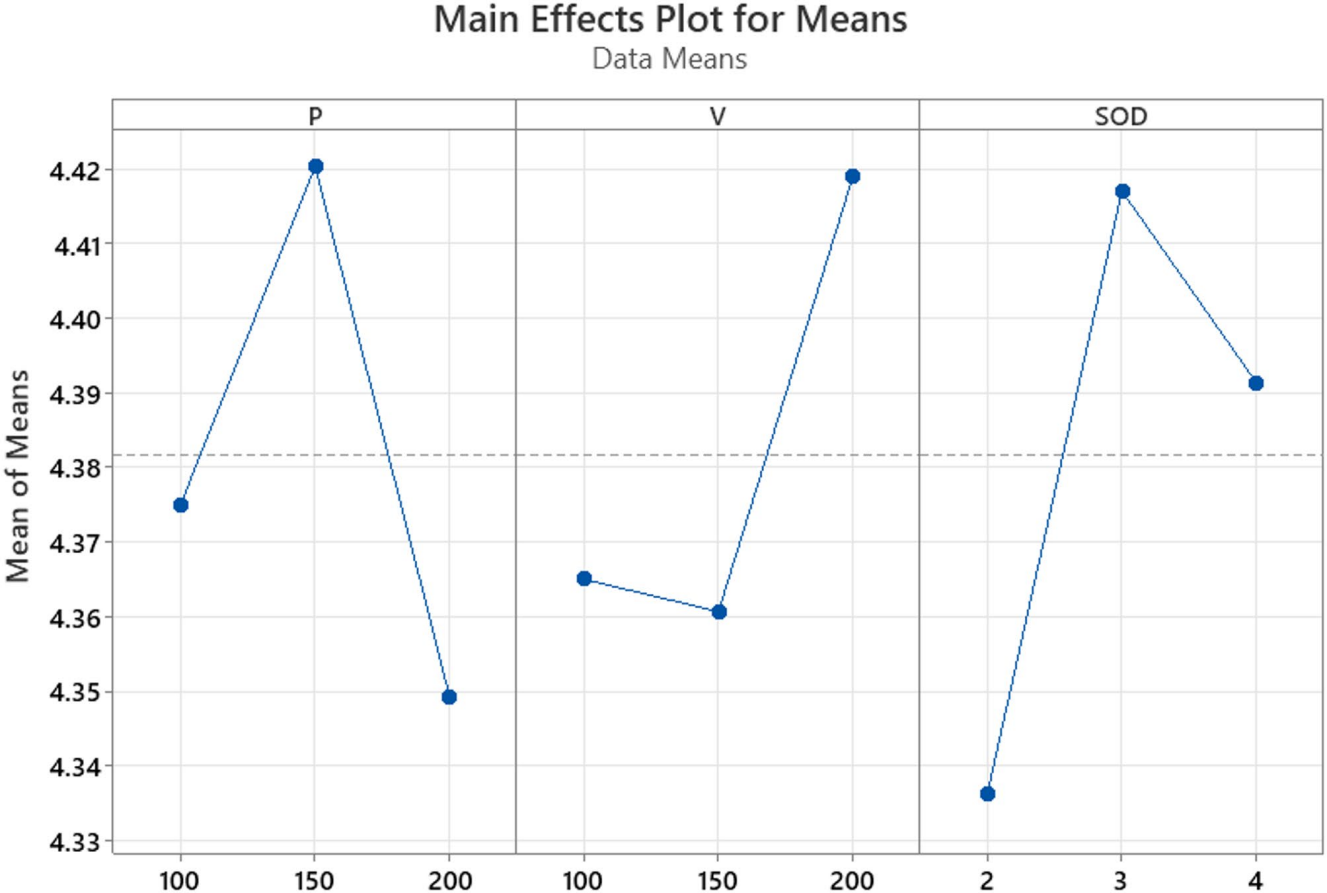
R_a_ main effect plots.

The S/N ratios for R_a_ across the experimental trials ranged from − 13.0023 to − 12.6410. Experiment 1, conducted at low waterjet pressure (100 MPa), low traverse speed (100 mm/min), and minimum stand-off distance (2 mm), yielded the highest S/N ratio (–12.6410), corresponding to the lowest recorded R_a_ of 4.286 μm. In contrast, experiment 4, which employed medium pressure (150 MPa), low traverse speed, and medium SOD (4 mm), exhibited the lowest S/N ratio (–13.0023), indicating the poorest surface finish among all trials.

Among the three process parameters, traverse speed emerged as the most influential factor affecting R_a_. Lower traverse speeds facilitated extended interaction between the abrasive particles and the workpiece surface, promoting uniform and controlled erosion. This resulted in reduced surface irregularities and consequently a smoother surface.

The role of waterjet pressure was also significant. A pressure level of 100 MPa was found to be optimal for achieving a fine surface finish. Higher pressures (≥ 150 MPa) increased abrasive particle velocity and turbulence, resulting in aggressive material removal, overcutting, and micro-pitting factors detrimental to surface quality.

Stand-off distance further influenced the cutting dynamics. A shorter SOD of 2 mm enhanced the jet’s focus and energy concentration at the cutting interface, minimized jet divergence, and improved machining accuracy. Longer SODs led to increased jet dispersion and reduced energy density, thereby degrading the surface finish.

The trends are further supported by the Main Effects Plot for Means, as illustrated in Fig. 8. This plot displays the average R_a_ values for each level of the parameters: P, V, and SOD. A steep slope in the plot indicates a greater influence of a parameter on the response. The plot clearly shows that V has the steepest gradient, highlighting its significant impact on surface roughness. P also demonstrates notable variation across its levels, with the lowest R_a_ values observed at 100 MPa. In contrast, SOD shows moderate variation, with the best surface finish achieved at a distance of 2 mm.

The nonlinear behaviour of surface roughness in AWJM arises from the combined effects of abrasive energy, polymer matrix deformation, and composite filler interaction. At moderate water pressures, increased particle velocity enhances cutting, reducing R_a_. However, very high pressures can cause irregular erosion and micro-tearing due to turbulence, increasing R_a_. At low traverse speeds, longer jet exposure can lead to smoother surfaces, but excessive exposure causes over-erosion, increasing roughness. At higher speeds, insufficient cutting time leads to poor surface finish. Stand-off distance also affects jet coherence too small causes splash-back; too large reduces jet focus both increasing R_a_. The presence of ZrO₂-coated MWCNTs alters heat dissipation and erosion resistance, contributing to uneven surface profiles^38^.

Experiment 1 (P1–V1–SOD1) revealed the ideal machining conditions for achieving optimal surface roughness. It highlighted the importance of maintaining low pressure, a slow traverse speed, and minimal SOD to enhance jet coherence and control erosion. These factors worked together to produce a narrower kerf and a superior surface finish, demonstrating their suitability for the precise machining of polymer nanocomposites using AWJM.

### Effect of process parameters on Kerf taper (KT)

Kerf taper (KT), defined as the angular deviation between the entry and exit widths of the cut, is a critical quality attribute in AWJM, particularly for precision applications. It is primarily influenced by jet momentum decay and spatial dispersion as the jet penetrates the workpiece. The S/N ratio analysis for KT, based on the smaller-the-better criterion, revealed significant variation across process conditions, with values ranging from 18.2131 (Experiment 3) to 26.5072 (Experiment 8). A higher S/N ratio indicates a lower kerf taper and therefore superior dimensional precision.

**Fig. 9:**
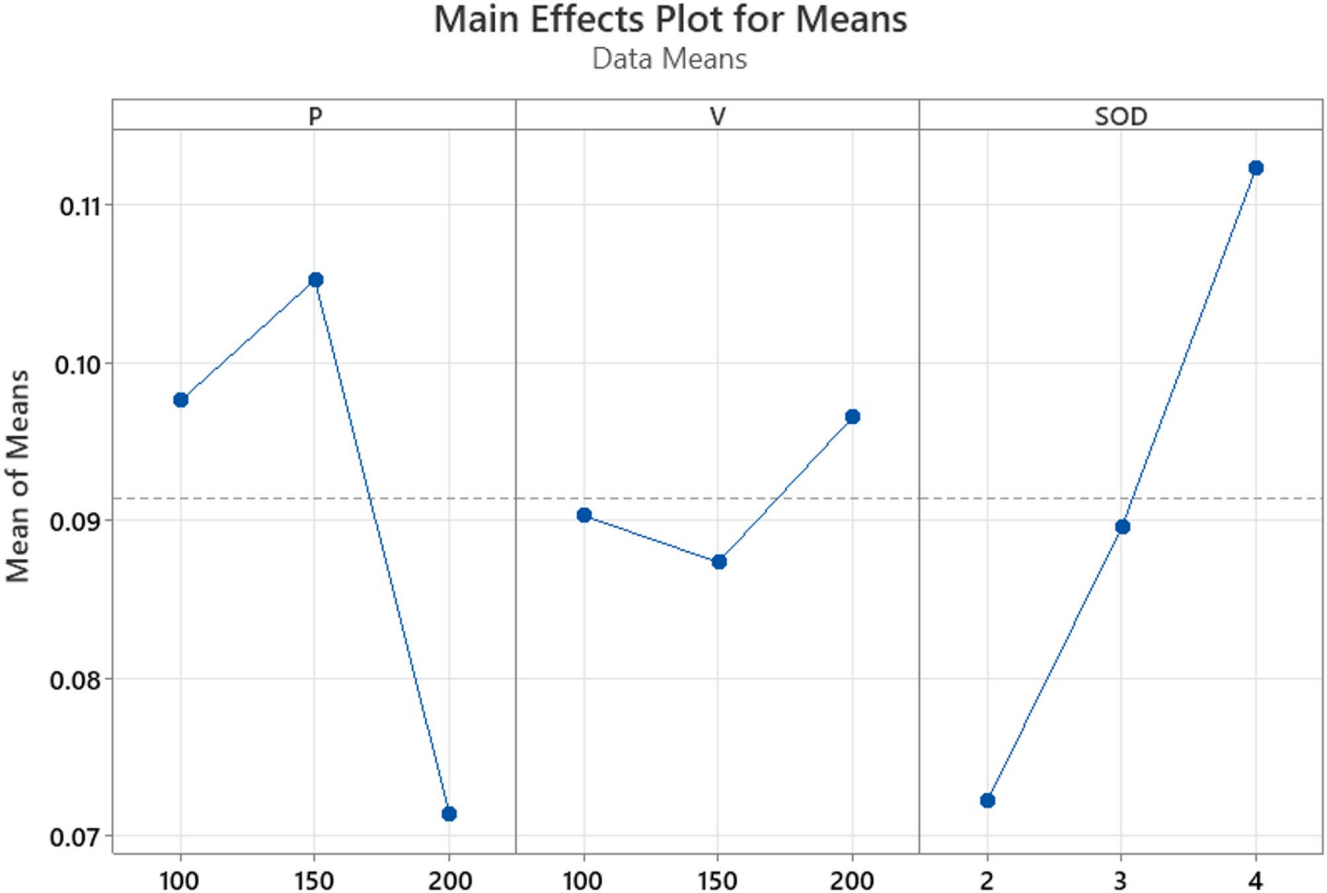
KT main effect plots.

Experiment 8, which employed high waterjet pressure (200 MPa), medium traverse speed (150 mm/min), and low stand-off distance (2 mm), resulted in the lowest measured kerf taper of 0.04727 radians, corresponding to the highest S/N ratio (26.5072). The enhanced performance under these conditions can be attributed to synergistic effects among the process parameters.

Waterjet pressure emerged as the most influential factor affecting KT. Higher pressure levels significantly improved jet collimation and penetration capability by increasing the kinetic energy of abrasive particles. This reduced jet divergence during depth wise penetration and minimized angular deviation of the cut walls. In contrast, lower pressures were associated with broader kerf profiles due to insufficient particle energy and premature jet deflection.

The traverse speed also played a vital role in governing the jet-material interaction dynamics. A moderate traverse speed of 150 mm/min offered an optimal balance between erosion time and jet stability. At this speed, the jet retained sufficient coherence and energy to sustain a uniform cutting path, avoiding both undercutting (common at very high speeds) and washout effects (seen at excessively low speeds). Stand-off distance was another critical parameter. A short SOD of 2 mm minimized the jet’s expansion before impact, thereby preserving its focus and energy density at the workpiece surface. This led to a straighter and more uniform kerf profile, with reduced divergence at the exit plane.

The findings are supported by the Main Effects Plot for Means shown in Fig. 9. This plot illustrates how the average KT values respond to different levels of the input factors: P, traverse V, and SOD. The steepness of the curve indicates the level of influence each factor has. P demonstrates the most pronounced slope, confirming its significant effect on kerf taper. KT decreases sharply as pressure increases from 150 to 200 MPa, indicating that higher pressures lead to improved kerf straightness. Stand-off distance also shows a steep increasing trend, suggesting that a greater SOD results in increased kerf taper due to the dispersion and defocusing of the jet over longer distances. On the other hand, V displays a less steep and somewhat parabolic profile, reflecting its moderate influence. The lowest mean KT is observed at the middle level of traverse speed (150 mm/min), which aligns with the optimized erosion dynamics.

The nonlinear variation of kerf taper with AWJM parameters is due to the complex interplay of jet dynamics and material behaviour. At moderate water pressures, improved jet penetration reduces KT, but at very high pressures, increased turbulence leads to top overcutting and higher KT. Traverse speed affects jet exposure time too slow leads to top-edge widening, while too fast reduces bottom erosion, both increasing KT. Stand-off distance influences jet focus; low SOD improves accuracy, but too low or too high can increase KT due to clogging or jet dispersion. Additionally, the ZrO₂-MWCNT reinforcement increases hardness, creating variable erosion resistance through the thickness, further contributing to kerf taper irregularities^39^.

These observations collectively demonstrate that kerf taper is highly sensitive to P and SOD, with traverse speed playing a moderating role. Experiment 8 (P3–V2–SOD1) was identified as the most effective combination for minimizing kerf taper, making it the optimal parameter setting for achieving high dimensional accuracy in the AWJM of the investigated polymer nanocomposite.

### Effect of process parameters on material removal rate (MRR)

Material removal rate (MRR) is a crucial performance metric in AWJM, as it reflects the efficiency of the cutting process in terms of material disintegration. The S/N ratio analysis for MRR, based on the larger-the-better criterion, exhibited significant variation across the experiments, with values ranging from − 40.7571 (Experiment 3) to − 33.6713 (Experiment 1). A higher S/N ratio indicates a more efficient material removal process.

**Fig. 10:**
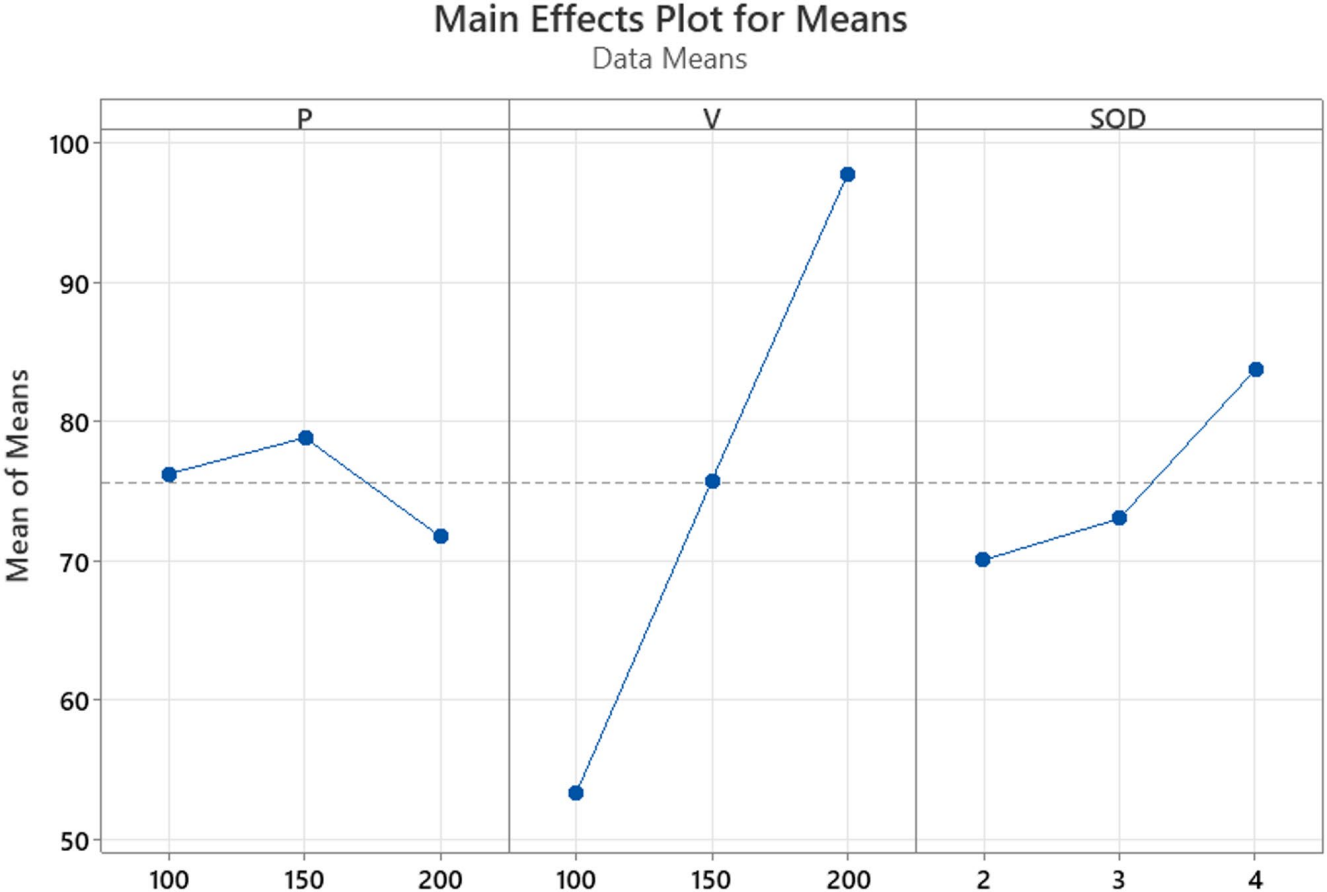
MRR Main effect plots.

Experiment 1, which employed low waterjet pressure (100 MPa), low traverse speed (100 mm/min), and minimal stand-off distance (2 mm), resulted in the highest S/N ratio (–33.6713) and the maximum MRR of 48.25 mm/min. This outcome suggests that a controlled erosion regime at lower speeds and pressures leads to more efficient material removal by preventing excessive energy loss and optimizing the jet-material interaction.

In contrast, experiment 3, which utilized high pressure (200 MPa), high traverse speed (200 mm/min), and a high stand-off distance (4 mm), resulted in the lowest S/N ratio (–40.7571), indicating the poorest material removal performance. Despite the high pressure, the excessive traverse speed and stand-off distance led to significant jet dispersion and energy loss, reducing cutting efficiency. At higher pressures and speeds, the abrasive particles are prone to scattering, forming a turbulent plume, which weakens jet-material coupling and lowers the effectiveness of material removal.

The influence of waterjet pressure on MRR is multifaceted. While higher pressures generally increase material removal by enhancing particle kinetic energy, excessive pressure can lead to over-penetration and excessive jet divergence, thereby reducing cutting efficiency. Similarly, high traverse speeds contribute to faster cutting, but at the cost of reduced jet coherence and focus, which diminishes MRR. On the other hand, lower traverse speeds allow for more controlled particle interaction with the workpiece, leading to higher material removal efficiency.

Stand-off distance also plays a pivotal role in determining MRR. A short SOD enhances jet focus and energy density at the cutting interface, ensuring more efficient material removal. Conversely, a larger SOD leads to increased jet dispersion, diminishing cutting power and lowering MRR. However, beyond a certain pressure level, the jet becomes highly turbulent and may disperse before optimally impacting the surface, especially when the SOD is non-negligible. This reduces the effective energy density at the target, causing a drop in MRR^40^.

These findings are further substantiated by the Main Effects Plot in Fig. 10 for Means V exhibits the steepest slope, indicating it is the most dominant factor influencing MRR. A clear upward trend is seen as the speed increases from 100 to 200 mm/min, aligning with the fact that higher traverse speeds remove more material per unit time though only to an optimal extent before losses due to dispersion set in. SO) also shows a consistent increasing trend, confirming that higher SOD correlates with increased MRR in this study possibly due to broader impact areas at the cost of surface finish. P has a relatively moderate influence, showing a non-linear trend with a peak at 150 MPa. This suggests an optimal pressure range exists, beyond which the efficiency gains taper off or reverse due to jet instability.

In conclusion, experiment 1 (P1–V1–SOD1) provided the optimal conditions for maximizing MRR, with a combination of low pressure, low traverse speed, and minimal SOD ensuring coherent jet flow and effective material removal. These findings suggest that, for optimal MRR in AWJM of polymer nanocomposites, a controlled erosion regime with moderate pressures and minimal stand-off distance is preferred.

### GRA

The normalized values, presented in Table 6, reveal significant variability in the performance of different experimental runs. Experiment 1 attained a normalized R_a_ value of 1.000, indicating the lowest actual surface roughness among all trials, thereby suggesting superior surface quality. However, its MRR value was 0.000, reflecting the least material removal and thus suboptimal machining efficiency. In contrast, experiment 3 yielded the maximum MRR (normalized value = 1.000) but registered poor surface quality and kerf control, as seen from its minimal normalized values for R_a_ (0.093) and KT (0.000).

**Table 6:** Normalized experimental data.

Experiment no	Normalized experimental data		
	*R*_a_ (mm)	KT (radians)	MRR (mm^3^/min)
1	1	0.603307	0
2	0.43956	0.398427	0.381797
3	0.093407	0	1
4	0	0.283603	0.136121
5	0.472527	0.008796	0.636123
6	0.313187	0.404444	0.736715
7	0.697802	0.404432	0.11481
8	0.857143	1	0.338859
9	0.401099	0.636735	0.704666

**Table 7:** Grey relation coefficient and grade values.

Experiment no	Deviation sequence			Grey relation coefficient			Grade	Rank
	*R*_a_ (mm)	KT (radians)	MRR (mm^3^/min)	*R*_a_ (mm)	KT (radians)	MRR (mm^3^/min)		
1	0	0.396693	1	1	0.557604	0.333333	0.630313	2
2	0.56044	0.601573	0.618203	0.471503	0.453896	0.447146	0.457515	8
3	0.906593	1	0	0.355469	0.333333	1	0.562934	3
4	1	0.716397	0.863879	0.333333	0.41105	0.366601	0.370328	9
5	0.527473	0.991204	0.363877	0.486631	0.3353	0.578786	0.466906	7
6	0.686813	0.595556	0.263285	0.421296	0.456389	0.655064	0.510916	5
7	0.302198	0.595568	0.88519	0.623288	0.456384	0.360961	0.480211	6
**8**	**0.142857**	**0**	**0.661141**	**0.777778**	**1**	**0.430611**	**0.73613**	**1**
9	0.598901	0.363265	0.295334	0.455	0.579197	0.628666	0.554288	4

Optimized values are in [bold].

Experiment 8 is noteworthy for achieving the maximum normalized KT value of 1.000, implying the least kerf taper and hence high geometrical precision. Moreover, it exhibited a relatively high normalized R_a_ (0.857) and moderate MRR (0.339), indicating balanced performance across multiple metrics. Likewise, experiment 6 demonstrated intermediate values for all three responses (R_a_ = 0.313, KT = 0.404, MRR = 0.737), suggesting a favorable compromise between surface quality, dimensional accuracy, and productivity.

The observed trends affirm that no single experimental condition simultaneously optimizes all responses in isolation. This underscores the necessity of adopting a GRA-based multi-criteria decision-making framework.

A close examination of Table 7 reveals that experiment 8 achieved the highest GRG of 0.7361 and was consequently ranked first. This can be attributed to its superior performance in minimizing kerf taper (KT = 1.000), along with favourable R_a_ and MRR values. Experiment 1 followed closely with a GRG of 0.6303 and was ranked second, due to its outstanding surface roughness (R_a_ = 1.000) and moderate kerf control. However, its MRR was the lowest (normalized = 0.000), indicating a trade-off between quality and productivity. Experiment 3, which achieved the best MRR (normalized = 1.000), was ranked third with a GRG of 0.5629, despite poor surface and kerf characteristics. This reflects the sensitivity of the GRA method in capturing multi-objective trade-offs. Experiment 4, on the other hand, showed the lowest GRG (0.3703) and was ranked ninth, indicating poor combined performance across all responses.

Overall, the GRG-based ranking confirms the effectiveness of GRA in identifying optimal process parameters that offer a balanced performance across multiple conflicting quality criteria. Notably, trials with extreme performance in a single metric but poor results in others (e.g., Experiment 3) were outperformed by those with more consistent, moderately good values across all responses (e.g., Experiments 1 and 8). This analysis demonstrates that for the 3 wt% ZrO_2_-MWCNT/HDPE composite, experiment 8 represents the most optimal parameter setting, achieving a high-quality machined surface with minimal taper and a reasonably high MRR. This balanced performance is critical in precision polymer composite machining applications, where both surface integrity and productivity are essential.

## Conclusion

This study systematically examined the machinability of HDPE reinforced with 3 wt% ZrO₂ coated MWCNTs using AWJM. The Taguchi-Grey Relational Analysis method was employed to optimize the process parameters for surface roughness, kerf taper, and material removal rate. The experimental results showed that traverse speed had the most significant impact on R_a_. Lower speeds reduced surface irregularities, achieving a minimum surface roughness of R_a_ = 4.286 μm due to an extended abrasive-workpiece interaction. In contrast, waterjet pressure was the primary factor influencing KT reduction. At 200 MPa, the enhanced jet coherence led to the lowest taper angle by improving particle momentum retention.

The stand-off distance had a critical effect on MRR, with shorter distances maximizing energy density and erosion efficiency. Multi-objective optimization using GRA identified the optimal combination of parameters: 200 MPa waterjet pressure, 150 mm/min traverse speed, and 2 mm SOD. This combination achieved the highest grey relational grade of 0.7361 by balancing minimal R_a_ and KT while maximizing MRR. The synergistic effect of ZrO₂-MWCNT reinforcement paired with carefully controlled AWJM parameters highlights the composite’s suitability for precision machining. Additionally, the Taguchi-GRA framework offers a robust methodology for optimizing multiple responses in polymer nanocomposite processing. Future research could investigate dynamic parameter adaptation or hybrid reinforcements to further improve machinability.

## Data Availability

The datasets generated and/or analysed during the current study are available from the corresponding author on reasonable request.
